# Is disrupted sleep a risk factor for Alzheimer’s disease? Evidence from a two-sample Mendelian randomization analysis

**DOI:** 10.1093/ije/dyaa183

**Published:** 2020-05-11

**Authors:** Emma L Anderson, Rebecca C Richmond, Samuel E Jones, Gibran Hemani, Kaitlin H Wade, Hassan S Dashti, Jacqueline M Lane, Heming Wang, Richa Saxena, Ben Brumpton, Roxanna Korologou-Linden, Jonas B Nielsen, Bjørn Olav Åsvold, Gonçalo Abecasis, Elizabeth Coulthard, Simon D Kyle, Robin N Beaumont, Jessica Tyrrell, Timothy M Frayling, Marcus R Munafò, Andrew R Wood, Yoav Ben-Shlomo, Laura D Howe, Deborah A Lawlor, Michael N Weedon, George Davey Smith

**Affiliations:** 1 MRC Integrative Epidemiology Unit, at the University of Bristol, Bristol, UK; 2 Population Health Sciences, University of Bristol Medical School, Bristol, UK; 3 Genetics of Complex Traits, University of Exeter Medical School, Exeter, UK; 4 Center for Genomic Medicine, Massachusetts General Hospital, Harvard Medical School, Boston, MA, USA; 5 Program in Medical and Population Genetics, Broad Institute, Cambridge, MA, USA; 6 Department of Anesthesia, Critical Care and Pain Medicine, Massachusetts General Hospital, Boston, MA, USA; 7 Division of Sleep and Circadian Disorders, Brigham and Women’s Hospital, Harvard Medical School, Boston, MA, USA; 8 Department of Public Health and Nursing, K.G. Jebsen Center for Genetic Epidemiology, Norwegian University of Science and Technology, Trondheim, Norway; 9 Department of Thoracic Medicine, St Olavs Hospital, Trondheim University Hospital, Trondheim, Norway; 10 Department of Internal Medicine, Division of Cardiovascular Medicine, University of Michigan, Ann Arbor, MI, USA; 11 Department of Endocrinology, St. Olavs Hospital, Trondheim University Hospital, Trondheim, Norway; 12 Translational Health Sciences, University of Bristol Medical School, Bristol, UK; 13 Sleep and Circadian Neuroscience Institute, Nuffield Department of Clinical Neurosciences, University of Oxford, Oxford, UK; 14 UK Centre for Tobacco and Alcohol Studies, School of Psychological Science, University of Bristol, Bristol, UK

**Keywords:** Sleep, Alzheimer’s disease, dementia, Mendelian randomization, causal inference

## Abstract

**Background:**

It is established that Alzheimer’s disease (AD) patients experience sleep disruption. However, it remains unknown whether disruption in the quantity, quality or timing of sleep is a risk factor for the onset of AD.

**Methods:**

We used the largest published genome-wide association studies of self-reported and accelerometer-measured sleep traits (chronotype, duration, fragmentation, insomnia, daytime napping and daytime sleepiness), and AD. Mendelian randomization (MR) was used to estimate the causal effect of self-reported and accelerometer-measured sleep parameters on AD risk.

**Results:**

Overall, there was little evidence to support a causal effect of sleep traits on AD risk. There was some suggestive evidence that self-reported daytime napping was associated with lower AD risk [odds ratio (OR): 0.70, 95% confidence interval (CI): 0.50–0.99). Some other sleep traits (accelerometer-measured ‘eveningness’ and sleep duration, and self-reported daytime sleepiness) had ORs of a similar magnitude to daytime napping, but were less precisely estimated.

**Conclusions:**

Overall, we found very limited evidence to support a causal effect of sleep traits on AD risk. Our findings provide tentative evidence that daytime napping may reduce AD risk. Given that this is the first MR study of multiple self-report and objective sleep traits on AD risk, findings should be replicated using independent samples when such data become available.

**Key Messages**

- It is currently not clear whether disrupted sleep is a causal risk factor for Alzheimer’s disease; current observational associations are likely biased by reverse causation and confounding.
- We employ the largest genome-wide association studies of multiple sleep traits in Mendelian randomization analyses, to examine whether sleep disruption is causally linked to Alzheimer’s disease risk.
- We find very limited evidence of link between disrupted sleep and risk of Alzheimer’s disease. However, there is tentative evidence that daytime napping may be protective.

## Introduction

Alzheimer’s disease (AD) has been estimated to affect 47 million people worldwide and the prevalence is expected to double in the next 20 years.^1^ Current treatments are unable to reverse or delay progression of the disease, highlighting the importance of prevention. Identifying causal, modifiable risk factors is crucial for developing successful prevention strategies. It is well established that patients with AD experience sleep disruption (e.g. shorter duration, greater fragmentation).^2^ However, it remains unknown whether disruption in the quantity, quality or timing of sleep is a causal risk factor for the onset of AD.

Various sleep parameters have previously been suggested as potential risk factors for AD,^3–6^ but research to date has yielded inconsistent findings. Authors of the recent *Lancet* commission on ‘Dementia prevention, intervention, and care’, did not include sleep in their calculations of population-attributable fractions of the most ‘potent’ dementia risk factors (despite acknowledging sleep as a potentially important risk factor) due to the absence of systematic reviews or enough consistent, high-quality evidence.^3^ Inconsistencies in the sleep-AD literature may, at least in part, be explained by bias due to reverse causation. Most studies have been conducted in clinical populations (e.g. patients with mild cognitive impairment or early AD), making it difficult to rule out that associations are not due to sleep disruption as a result of accumulating AD pathology. Very few studies have been conducted in healthy (non-clinical) populations and, even those that have, tend to include older participants (i.e. mid- to-late life at baseline).^7^ AD has a long prodromal phase of up to 20 years.^8^ Thus, even in apparently healthy populations, measuring sleep in later life makes it difficult to rule out that those participants with sleep disruption are those with prodromal AD. Another potential explanation for the inconsistencies may be the considerable heterogeneity in existing study designs, which have examined various exposures (e.g. sleep duration,^9^ time spent in sleep stages,^10^ fragmentation,^11^ insomnia^12^ and frequency and duration of daytime napping^13^ measured both subjectively and objectively) and outcomes (e.g. cognitive function at a single time point,^14^ cognitive decline over time,^15^ mild cognitive impairment,^16^ AD diagnoses^16^ and putative AD biomarkers such as amyloid-beta and tau^13^). Finally, the majority of studies conducted to date are observational, meaning confounding is a plausible explanation for the findings.

Mendelian randomization (MR) is a method that uses genetic variants as instrumental variables for environmental exposures, to estimate the causal effect of an exposure on an outcome. Due to their random allocation at conception, genetic markers of a risk factor are largely independent of potential confounders that may otherwise bias the association of interest. They also cannot be modified by subsequent disease, thereby eliminating potential bias by reverse causation.^17^ Thus, MR is a useful tool for helping to establish whether sleep traits are causally related to risk of onset of AD, or whether associations observed to date are likely a result of bias by confounding and/or reverse causation. In this study, we aimed to establish whether both self-report and accelerometer-measured sleep traits have a causal effect on AD risk, using a two-sample MR design.^18^

## Methods

Methods for conducting two-sample MR analyses have been published previously.^18^ Briefly, two-sample MR provides an estimate of the causal effect of an exposure on an outcome, using independent samples to obtain the gene-exposure and gene-outcome associations, provided three key assumptions hold: (i) genetic variants are robustly associated with the exposure of interest (i.e. replicate in independent samples); (ii) genetic variants are not associated with potential confounders of the association between the exposure and the outcome; and (iii) there are no effects of the genetic variants on the outcome, independent of the exposure (i.e. no horizontal pleiotropy).^19^

### Data

**Figure 1:**
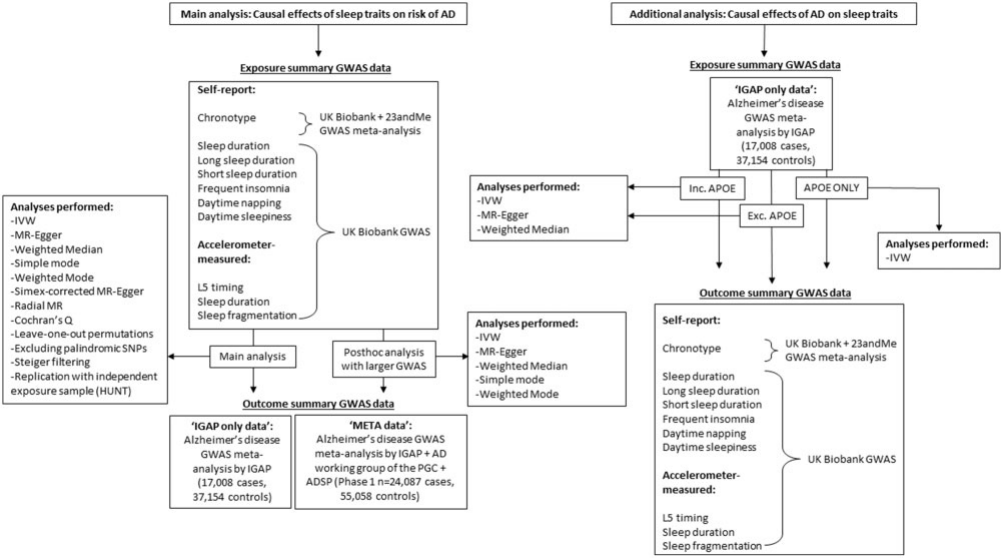
Flow chart detailing data sources for each analysis performed. AD, Alzheimer’s disease; GWAS, genome-wide association study; MR, Mendelian randomization; IGAP, International Genomics of Alzheimer’s Project; IVW, Inverse Variance Weighted; PGC, Psychiatric Genomics Consortium; ADSP, Alzheimer’s Disease Sequencing Project

Genome-wide association studies (GWAS) have been previously performed for seven self-reported measures of habitual sleep patterns: chronotype,^20^ sleep duration,^21^ long sleep duration,^21^ short sleep duration,^21^ frequent insomnia,^22^ excessive daytime sleepiness^23^ and daytime napping. GWAS have also previously been performed for three accelerometer-measured measures of sleep including timing of the least active 5 h of the day (L5 timing), nocturnal sleep duration and sleep fragmentation.^24^ Note that the assessment of accelerometer-derived sleep for up to 7 days per individual in UK Biobank was performed, on average, 5 years after the self-report sleep data were collected.^25^ Full details of each GWAS (e.g. cohorts included, ancestral groups, exclusion criteria, covariate adjustment etc.) are provided in Supplementary Table A, available as Supplementary data at *IJE* online. Table 1 provides a brief description of each of the sleep traits, the units in which they were measured, participant numbers, the number of approximately independent genome-wide significant (*P*< 5 x 10^-8^) loci identified and the F statistic. F statistics provide an indication of instrument strength^26^ and are a function of how much variance in the trait is explained by the set of genetic instruments being used, the number of genetic instruments being used and the sample size. F  10 indicates that the analysis is unlikely to suffer from weak instrument bias.^27^ For the outcome, we used the large-scale GWAS meta-analysis of AD, conducted by the International Genomics of Alzheimer’s Project (IGAP) (*n* = 17 008 AD cases and 37 154 controls).^28^ Ethics approval was obtained by the original GWAS studies. Figure 1 is a flow chart describing the data sources for each set of analyses performed.

**Table 1:** Description of the sleep genome-wide association studies (GWAS) included in the two-sample Mendelian randomization analyses

	Trait definition (units)	**Participants, *n*(cases/controls** **for binary traits)**	Loci identified, *n*, in GWAS	F statistic
Self-report measures				
Chronotype^20^	Whether a person identifies as being a ‘morning person’ or an ‘evening person’ (ordered categorical variable of definitely a morning person, more a morning than an evening person, do not know, more an evening than morning person and definitely an evening person)^a^	449 734	351	33.1
Sleep duration^21^	Average number of hours slept in 24 h, including naps (continuous variable, hours)	446 118	78	39.6
Short sleep duration^21^	Person has an average of 6 h or less per night vs 7-8 h per 24 h (binary variable of yes/no)	411 934 (106 192/305 742)	27	25.6
Long sleep duration^21^	Person has an average of 9 h or more per night vs 7-8 h per 24 h (binary variable of yes/no)	339 926 (34 184/305 742)	8	30.6
Frequent insomnia^22^	Person has trouble falling asleep at night or wakes up in the middle of the night (binary variable of usually vs never/rarely)	453 379 (131 480/321 899)	48	41.7
Excessive daytime sleepiness^23^	Person dozes off or falls asleep during the day without meaning to (ordered categorical variable of never or rarely, sometimes, often and all the time)	452 071	37	42.3
Daytime napping	Person naps during the day (ordered categorical variable of never, sometimes, usually)	452 633	112	46.1
Accelerometer measures				
L5 timing^24^	Timing of the least active 5 h of the day (continuous variable of hours elapsed since previous midnight; provides indication of phase of most restful hours with later times indexing greater tendency towards ‘eveningness’)	85 205	6	55.3
Sleep duration^24^	Average number of hours of nocturnal sleep per night (continuous variable, hours)	84 810	11	52.1
Sleep fragmentation^24^	The average number of nocturnal sleep episodes separated by at least 5 min of wakefulness per night (continuous variable, number of episodes)	84 810	21	38.6

a Note that in the original chronotype GWAS, categories were ordered from more ‘eveningness’ to more ‘morningness’. In this analysis, to ensure that the ordinal chronotype variable correlated positively with the accelerometer-measured measure of L5 timing, single nucleotide polymorphism (SNP)-exposure coefficients for chronotype were reordered from more ‘morningness’ to more ‘eveningness’ (where ‘definitely a morning person’ is the reference category).

## Statistical analysis

### Estimating the causal effects of the sleep traits on risk of Alzheimer’s disease

**Figure 2:**
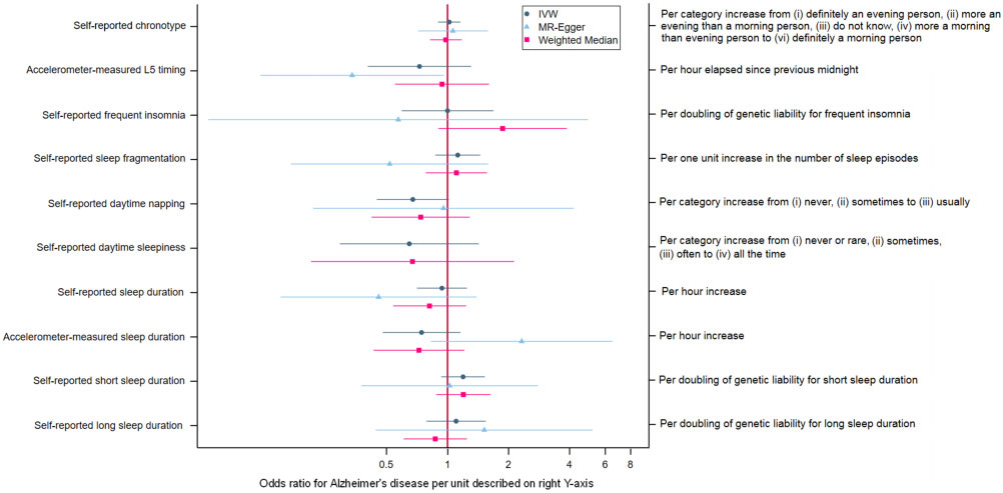
Associations of sleep traits with Alzheimer’s Disease. Note that the MR Egger estimate for the effect of daytime sleepiness was not plotted due to imprecision. IVW, Inverse Variance Weighted

Full details of the harmonization procedure can be found in the online supplement. Supplementary Table B, available as Supplementary data at *IJE* online, shows the single nucleotide polymorphism (SNP) flow through the harmonization procedure. MR-Base (www.mrbase.org)^29^ was employed to perform all two-sample MR analyses. Effect estimates and corresponding standard errors of the genome-wide significant SNPs were extracted from each sleep GWAS and the AD GWAS. The SNP-exposure (sleep trait units detailed in Table 1 and Figure 2) and SNP-outcome [AD, in units of log odds ratios (ORs] coefficients were combined using an inverse-variance-weighted (IVW) approach to give an overall estimate of the causal effect across all SNPs included for each sleep trait. The estimator is a Wald ratio and is equivalent to a weighted regression of the SNP-outcome coefficients on the SNP-exposure coefficients with the intercept constrained to zero. The results of all analyses were converted to ORs for AD. For binary exposures (i.e. frequent insomnia, and long and short sleep duration), SNP-exposure coefficients were estimated using logistic regression and are therefore on the log odds scale. Causal effect estimates (i.e. ORs for AD) have been rescaled so that they are interpreted per doubling of genetic liability for the sleep trait, as recommended by Burgess *et al.*^30^ For ordered categorical exposures, SNP-exposure coefficients were estimated using linear regression, and causal effect estimates are interpreted per category increase in the sleep trait. For continuous exposures, SNP-exposure coefficients were estimated using linear regression, and causal effect estimates are interpreted per unit increase in the sleep trait (units detailed in Table 1 and Figure 2).

### Sensitivity analyses

A series of sensitivity analyses were conducted to check for violation of the key MR assumptions. The rationale and methodological details for each of these analyses are provided in the online supplement. The IVW method assumes no horizontal pleiotropy but will be unbiased if there is balanced horizontal pleiotropy.^19^ Thus, results from the IVW method were compared with those from MR-Egger^19^ and weighted median regressions^31^ which relax this assumption. The IVW method also assumes no measurement error in the gene-exposure association estimates (i.e. the NOME assumption).^19^ We assessed this using an adaptation of the I^2^ statistic^32^ (referred to as $I_{\text{GX}}^{2}$) which provides an estimate of the degree of regression dilution in the MR-Egger causal estimate due to uncertainty in the SNP-exposure estimates. Simulation extrapolation (SIMEX) was then used to adjust the MR-Egger estimate for this dilution.^33^ Heterogeneity (i.e. variability in causal estimates from different genetic variants) was assessed using Cochran’s Q statistic.^19^ Funnel plots were then generated to enable visual assessment of the extent to which pleiotropy is likely to be balanced (or directional) across the set of instruments used in each analysis. Radial MR was used to detect and remove any SNP outliers (i.e. those SNPs that contribute the most heterogeneity to Cochran’s Q, based on a multiple testing corrected *P*-value threshold). Leave-one-out permutations were conducted to assess the undue influence of potentially pleiotropic SNPs on the causal estimates.^34^ We checked that results were similar after excluding palindromic SNPs.^35^ Steiger filtering was performed to test that the hypothesized causal direction was correct for each SNP (i.e. that the genetic instruments influence the exposure first and then the outcome, through the exposure).^36^ Finally, we investigated potential bias due to ‘winner’s curse’ where the magnitude of the effect sizes for variants identified within a single discovery sample are likely to be larger than in the overall population, even if they are truly associated with the exposure. Assessment of winner’s curse was only possible for frequent insomnia and sleep duration, where the GWAS were replicated in independent samples [the Nord-Trøndelag Health Study (HUNT) and the Cohorts for Heart and Aging Research in Genomic Epidemiology (CHARGE), respectively]. Details of the replication samples and winners curse analyses are in the online supplement.

### Additional analyses

To examine whether the previously observed associations between AD (as an exposure) and sleep disruption (as an outcome)^2^ could be supported through applying MR analytical approaches, we tested whether genetic liability for AD was causally associated with the self-reported and accelerometer-measured sleep traits. We used 20 independent genome-wide significant AD SNPs identified in the IGAP AD GWAS meta-analysis (described previously)^28^ as genetic instruments in these analyses. As with the main analysis of sleep traits on AD risk, SNP-exposure and SNP-outcome coefficients were combined using an inverse-variance-weighted (IVW). MR-Egger, weighted median, radial MR and Steiger filtering were performed to assess potential violation of the MR assumptions. Analyses were conducted both with and without the apolipoprotein E (*APOE*) variant included, as *APOE* has been previously shown to be pleiotropic^37^ (which violates an MR assumption). We also examined associations of *APOE* (as a single genetic instrument) with the sleep traits. As AD is a binary exposure and SNP-exposure coefficients are on the log odds scale, causal estimates for the effect of AD on sleep traits are rescaled so that they are interpreted per doubling of genetic liability for AD.

It is worth noting that there are several important limitations to these analyses. First, the average age of AD diagnosis in the UK is around 75 years,^38^ with the neuropsychological effects of prodromal disease detectable up to 8 years preceding a diagnosis of mild cognitive impairment (i.e. around 10–12 years before AD diagnosis).^39^ Thus, the majority of UK Biobank participants are likely too young (average age at recruitment 56 years^40^) to have even prodromal disease at the time the sleep traits were measured. Examining the effect of AD on sleep traits in a relatively young, healthy population (when we hypothesize that any effects of AD on sleep traits will likely be a result of increasing pathological burden) may not yield reliable results. Second, there is ‘healthy selection’ into the UK Biobank; only approximately 5% of those invited to participate in the study accepted the invitation,^41^ and there is evidence that genetic liability to AD is associated with lower participation rates in the optional components of UK Biobank.^42^ This makes it less likely that participants with an existing AD diagnosis or undiagnosed prodromal disease would have been recruited into the study, and again, may induce bias in the causal effect estimates.

### *Post hoc* analyses

After the completion of the analyses for this paper, an updated AD GWAS meta-analysis was published^43^ including the aforementioned IGAP, the AD working group of the Psychiatric Genomics Consortium and the AD Sequencing Project (Phase 1; *n *= 24 087 cases and 55 058 controls compared with *n* = 17 008 AD cases and 37 154 controls for IGAP alone). We therefore conducted a sensitivity analysis to assess whether causal effects were consistent when using the newer GWAS. We a priori decided not to use summary statistics from the Phase 3 meta-analysis (which includes UK Biobank), but instead use the Phase 1 (which excludes UK Biobank) for two reasons: first, all sleep trait GWASs include the UK Biobank, meaning there would be significant overlap between the exposure and outcome samples in each MR analysis. This can yield biased causal effect estimates.^44^ Second, the Phase 3 AD GWAS meta-analysis includes only AD-by-proxy cases from the UK Biobank (i.e. no diagnosed cases). AD-by-proxy cases were defined as a positive response to the question ‘Has your mother or father ever suffered from Alzheimer’s disease/dementia’. There are several potential problems with this for MR analyses: participants defined as cases have not themselves been diagnosed with AD; the question does not specify Alzheimer’s disease but asks about any form of dementia; and finally, the question does not ask if family members were diagnosed by a doctor. For ease of the reader, we will henceforth refer to the main IGAP analyses as being in the ‘IGAP-only data’, and the additional analyses with the newer published GWAS as being in the ‘META data’.

## Results

### Main analyses in ‘IGAP-only data

Supplementary Table C, available as Supplementary data at *IJE* online, shows the phenotypic correlations between each of the sleep traits. Correlations between accelerometer measures have been published previously.^24^ Correlations were generally weak, ranging from r = -0.001 (between accelerometer-measured L5 timing and accelerometer-measured sleep duration) to r = -0.32 (between self-report frequent insomnia and self-report short sleep duration). It is also worth noting that correlations were weak between self-reported and accelerometer-measured sleep duration (*r *=* *0.15). This may reflect that accelerometer data in the UK biobank were collected between 2 and 9 years (mean 5 years)^24^ after baseline, when self-reported sleep measures were assessed. It may also reflect that self-reports of global sleep duration (vs daily self-reported) can be influenced by distress/affect.^45^

Figure 2 shows results for the analysis of the sleep traits on risk of AD. Full results can be found in Supplementary Table D, available as Supplementary data at *IJE* online. Point estimates for self-reported chronotype, insomnia and sleep duration were very close to or on the null. Both shorter and longer self-reported sleep duration and accelerometer-measured sleep fragmentation yielded positive estimates with AD risk, but were imprecisely estimated. There was some tentative evidence of a protective effect of self-reported daytime napping on AD risk, with odds of AD being 33% lower (95% CI: 55% lower to 2% higher) per category increase from ‘never’, ‘sometimes’ to ‘usually’ napping. However, this was again imprecisely estimated. Odds ratios for greater accelerometer-measured ‘eveningness’, longer accelerometer-measured sleep duration and self-reported daytime sleepiness were similar in magnitude to daytime napping, although again with wide confidence intervals.

### Sensitivity analyses in IGAP-only data

For all analyses, there was little evidence of directional pleiotropy from the MR-Egger regression intercepts (Supplementary Table E, available as Supplementary data at *IJE* online), and causal effect estimates from the MR-Egger and weighted median regressions generally agreed with those from the IVW regressions; in all cases there was substantial overlap between the confidence intervals for each estimate (Supplementary Table D, available as Supplementary data at *IJE* online). As expected, precision was less for MR-Egger (due to estimating both an intercept and slope in the MR-Egger regression as opposed to only a slope in the IVW regression) and weighted median (due to assuming only 50% of the instruments are valid). $I_{\text{GX}}^{2}$ statistics are provided in Supplementary Table F, available as Supplementary data at *IJE* online and SIMEX-adjusted MR-Egger estimates in Supplementary Table D. These estimates were consistent with regression dilution of the MR-Egger causal effect estimates due to measurement error in the SNP-exposure estimates. There was evidence of between-SNP heterogeneity in the self-reported chronotype and daytime napping, and accelerometer-measured L5-timing analyses (Supplementary Table G, available as Supplementary data at *IJE* online). However, these were not unduly asymmetrical in the funnel plots (Supplementary Figures A–C, available as Supplementary data at *IJE* online), suggesting that directional pleiotropy is unlikely to bias the effect estimates for these sleep traits. A total of three outliers were detected by radial MR for sleep fragmentation (rs12714404, rs429358 and rs4974697) and one for L5 timing (rs1144566). Point estimates for these two traits attenuated towards the null after removal of these outliers (Supplementary Table H, available as Supplementary data at *IJE* online). Results were similar after removing each SNP in turn in the leave-one-out permutations (Supplementary Figures D–M, available as Supplementary data at *IJE* online), suggesting that no single SNP was having undue influence on the overall causal effect estimates. Results were also similar when palindromic SNPs were excluded from the analyses (Supplementary Table I, available as Supplementary data at *IJE* online). Steiger filtering provided evidence that for each MR analysis, SNPs explained more variation in the sleep trait than in AD. Findings for the sleep duration and insomnia results were similar when repeating analyses using the available replication datasets (i.e. using SNP-exposure estimates from independent datasets) (Supplementary Table J, available as Supplementary data at *IJE* online), providing evidence that bias due to winner’s curse is unlikely.

### Additional analyses in IGAP-only data

Associations between genetic liability for AD and all sleep traits are provided in Supplementary Table K, available as Supplementary data at *IJE* online. All point estimates are interpreted per doubling of genetic risk for AD. There was little evidence that genetic liability for AD was associated with short sleep duration (<6 h vs 7–8 h per 24 h) or daytime sleepiness. Increased genetic liability for AD was associated with less frequent insomnia, reduced daytime napping and reduced sleep fragmentation. The effects were very small in magnitude (e.g. a doubling of genetic liability for AD was associated with, on average, a 0.4% lower risk of frequent insomnia). Point estimates for associations of genetic liability for AD with all other sleep traits were also very close to or on the null (e.g. a doubling of genetic liability for AD was associated with, on average, 0.3 min lower sleep duration). *P*-values were small, likely due to the strength of *APOE* as an instrument for AD. This is supported by the fact that, when using the full set of AD genetic instruments minus *APOE*, point estimates remained largely unchanged but confidence intervals were wider (Supplementary Table L, available as Supplementary data at *IJE* online). Given that these causal effect estimates are per doubling of genetic liability for AD, the magnitude of effect is very small and not likely to be clinically important. Results were similar when using *APOE* alone as an instrument for AD (i.e. excluding all other AD SNPs, Supplementary Table M, available as Supplementary data at *IJE* online). Results were comparable when using MR-Egger and weighted median regressions (Supplementary Table K) and after removal of outliers detected by radial MR (Supplementary Table N, available as Supplementary data at *IJE* online). Steiger filtering provided evidence that for each MR analysis, all AD SNPs explained more variation in AD than in the sleep trait. Given our concerns about selection bias in the UK Biobank for these analyses,^46^ we performed a *post hoc* analysis to assess whether causal effect estimates were comparable when using a different outcome sample. Methods for these analyses are provided in the online supplement. We tested the association between genetic liability for AD and frequent insomnia in *n* = 62 533 participants from the Nord-Trøndelag Health Study (HUNT).^47^ HUNT is a less selected sample with over 60% response rate (compared with <5% for UK Biobank). Results were comparable to the main analyses using UK Biobank, except that confidence intervals were wider (IVW odds ratio: 0.98 per doubling of genetic liability for AD, 95% CI: 0.95 to 1.01 in HUNT, vs IVW odds ratio: 0.99 per doubling of genetic liability for AD, 95% CI: 0.99 to 1.00 in the UK Biobank, Supplementary Table O, available as Supplementary data at *IJE* online).

### *Post hoc* analysis in the ‘META data’

**Figure 3:**
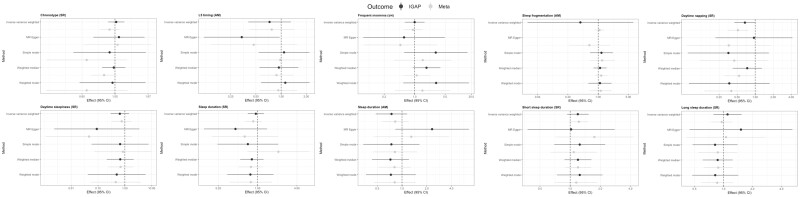
Comparing results for associations of sleep traits on risk of Alzheimer’s disease (AD) when using the International Genomics of Alzheimer’s Project (IGAP) genome-wide association study (GWAS) alone (*n* = 17 008 AD cases and 37 154 controls; IGAP) vs the GWAS meta-analysis of IGAP, the AD working group of the Psychiatric Genomics Consortium and the AD Sequencing Project (*n* = 24 087 cases and 55 058 controls: META data). Note that the MR Egger estimate for effect of accelerometer-measured sleep fragmentation using IGAP data was not plotted due to imprecision. MR, Mendelian randomization; AM, accelerometer-measured; SR, self-report

Causal effect estimates for associations of sleep traits on risk of AD were very similar when using the largest meta-analysis GWAS for AD, typically with more precision around the causal estimates (Figure 3). There was consistent evidence of a protective effect of daytime napping on Alzheimer’s risk, with odds of AD being 36% lower (95% CI: 11% to 45%) per category increase from ‘never’, ‘sometimes’ to ‘usually’ napping in the IVW analysis. Effects were consistent across several pleiotropy-robust methods, including MR Egger and weighted median approaches.

## Discussion

We have used the largest genome-wide association studies available of self-report and accelerometer-measured sleep traits and diagnosed Alzheimer’s disease, to provide evidence on possible causal relationships between them. Overall, based on both the IGAP-only data and the META data presented in this study, we found very little evidence to support a causal link between these sleep traits and risk of AD, except for some suggestive evidence that daytime napping may be associated with lower risk of AD. It is worth noting that odds ratios for accelerometer-measured ‘eveningness’, accelerometer-measured sleep duration and self-reported daytime sleepiness were similar in magnitude to daytime napping, but that confidence intervals were too wide to draw any meaningful conclusions (indicating that power is likely a key limitation here).

We found increased genetic liability for AD was associated with less frequent insomnia, reduced daytime napping and reduced sleep fragmentation. However, it is important to note that point estimates were very close to the null, and given that they are per doubling of genetic liability for AD, are unlikely to be clinically important. Confidence intervals around these point estimates were likely narrow due to the inclusion of *APOE* as a genetic instrument for AD (as it is strongly and robustly associated with a 3- to 15-fold increase in AD risk^43^); confidence intervals were much wider when we excluded this instrument. Although these associations are in the opposite direction to what we expected, these results should be treated with caution because of the potential for selection bias within the UK Biobank (which can cause spurious associations^46^) where participants are relatively young and healthier than the general population. In light of this and based on our largely null results, we would not rule out reverse causation as a potential explanation for previous findings in observational studies.

We looked only at frequency (not duration) of daytime napping, as information on duration of naps is not currently available in the UK Biobank. Previous studies have reported both positive and negative outcomes observed in relation to napping, and there is evidence that the duration may be particularly important (with shorter naps being beneficial, and longer naps being detrimental for various health outcomes including cardiovascular risk, cognitive impairment and memory consolidation).^48^^,^^49^ Most studies to date linking daytime napping to poor health outcomes have been done in elderly populations,^50^^,^^51^ making it difficult to rule out that (in those studies) daytime napping is not merely a result of underlying disease, rather than being a cause of the poor outcomes. In addition, it is often not possible in those studies to separate whether it is poor nocturnal sleep or the consequential daytime napping that is associated with the adverse outcomes. Conversely, there is some evidence that daytime naps offer a variety of benefits including memory consolidation^52^ and improvements in subsequent learning,^53^ executive functioning and emotional processing,^54^ all of which are impaired in AD.^55^ There is also evidence that short, restorative naps (<60 min in duration) may reduce the rate of cardiovascular disease and low-grade inflammation.^49^ Thus, daytime napping (before the onset of preclinical disease) may potentially serve as a useful compensatory mechanism for poor nocturnal sleep, enabling the brain to carry out tasks it was unable to complete during the night (for example, due to fragmented or short sleep).

There have been three twin-studies examining the relationship between sleep disruption and dementia risk, which may help mitigate bias due to confounding.^56–58^ However, two studies acknowledge that they are underpowered and include the null value,^56^^,^^57^ making it difficult to draw any inferences about causality, and one study included participants over age 65 years (with most being over age 70 years), making it plausible that estimates are biased by reverse causation.^58^ A recent MR study examined the causal effects of sleep duration on various dementia and cognitive function outcomes. Using standard MR methods, greater sleep duration was associated with slower reaction times and more errors in visual memory (with both outcomes measured as continuous variables), but there was no robust evidence of causal effects on reaction time or visual memory (both binary variables derived from the standardized regression-based method), all-cause dementia or, similar to our study, Alzheimer’s disease. Observing no associations with any of the binary outcomes may be due to a lack of power, as in our study. The authors also explored possible non-linearity in a one-sample framework, by generating three subgroups based on the residuals of sleep duration after adjustment on genetic instruments. This method explores the linear effect of sleep duration within each of these subgroups, and tests for evidence of a difference in associations (interactions) across them. They found evidence that associations with poorer visual memory and reaction time were stronger for both the lower and the higher duration subgroup than the middle one. However, it is worth noting that this method is limited for assessing non-linearity (as it is only really possible to obtain three strata) and may induce collider bias in cases where data are not measured continuously (as with self-report sleep duration in UK Biobank, which is measured categorically).^59^

### Strengths and limitations

To our knowledge, this is the first study to examine the causal effect of various sleep traits on risk of AD, using MR. We have both self-reported and accelerometer-assessed measures of sleep, allowing a comprehensive evaluation of various sleep parameters and a comparison across the two methods of assessment. We conducted a comprehensive series of sensitivity analyses to examine whether our results were robust to the various assumptions of MR or were likely to be biased by horizontal pleiotropy. We were also able to replicate some of our findings using independent exposure datasets (i.e. for self-report sleep duration and insomnia), and results were consistent, suggesting our findings are unlikely to be biased by winner’s curse. Findings were also largely consistent when we performed MR using summary statistics from a more recent GWAS meta-analysis of Alzheimer’s disease.^43^

There are, however, several limitations to our study. The first limitation is statistical power; confidence intervals were wide for most sleep traits (despite using the largest available GWASs), limiting our ability to draw meaningful conclusions for many associations. Identifying stronger instruments for these sleep traits may increase statistical power and enable us to estimate their causal effects on AD risk more precisely. The second limitation is that some SNPs may overlap across sleep traits. It would be of interest to perform multivariable MR analysis to fully delineate the independent effects of each of the sleep traits, but we would be underpowered to draw inferences from this with seven traits, some of which have very few genetic markers. This should, however, have little consequence for the interpretation of our results. Concern here would be about horizontal pleiotropy (an observed causal effect on the outcome that does not go via the exposure of interest, but another trait). Pleiotropy is important to consider in the context of non-null results, as we want to have some certainty that the pathway from gene to outcome goes via the exposure of interest. Our results are largely null, meaning there is little evidence of a direct effect of the exposure, or a pleiotropic effect, on the outcome. It is plausible that pleiotropy could act in the opposite direction to the true causal effect and bias estimates towards the null. However, this is unlikely given: (i) that none of the extensive sensitivity analyses performed suggest that pleiotropy is biasing effect estimates; and (ii) we observe no causal effects for any other (pleiotropic) sleep traits. Thus, pleiotropy is an unlikely explanation for our findings. The third limitation is that, for some sleep traits (e.g. self-report long-sleep duration and for accelerometer L5 timing and sleep duration), there were very few genome-wide significant SNPs identified in their respective GWASs (and thus available for use in our MR analyses). This made it difficult to examine potential directional horizontal pleiotropy using funnel plots for these traits. However, given that there was only evidence of heterogeneity for L5 timing, daytime napping and chronotype (and the chronotype and daytime napping showed no marked asymmetry in the funnel plot), horizontal pleiotropy is unlikely to explain our findings. Third, we did not correct for multiple testing because several of the sleep traits are correlated, but the results need to be interpreted in this light, and replication of our findings is required. Fourth, it is possible that the specific features of sleep that are implicated in the pathogenesis of AD (for example, disruption of slow-wave sleep) are not detectable using accelerometers or subjectively. Fifth, there may be a threshold effect of daytime napping by which shorter naps are beneficial and long, frequent naps may be detrimental. Previous studies have suggested this may be the case (particularly for cardiovascular risk, cognitive impairment and memory consolidation^49^) but we are unable to unpick these effects with current data in an MR framework. Sixth, most analyses were conducted using data from the UK Biobank, which may not be representative of the general population (due to selection into the study).^41^ That said, results were similar when using data from independent replication samples (including HUNT and CHARGE). All GWAS samples were also restricted to participants of European ancestry and therefore may not be generalizable to other populations. Finally, no sleep diaries were collected in the UK Biobank to identify time in bed and out of bed, which may introduce measurement error into some of the accelerometer measures. However, for the sleep data used in this study, times in and out of bed were estimated using a validated algorithm to determine the sleep period time window.^60^

### Conclusions

We found very limited evidence to support disrupted sleep as a causal risk factor for AD. Our results tentatively suggest that daytime napping may reduce AD risk. Given that this is the first MR study of multiple self-report and objective sleep traits on AD risk, findings should be replicated using independent samples when such data become available. Identifying stronger instruments for all sleep traits will be useful in more precisely estimating any causal effects on AD risk.

### Data availability statement

Most of the data in this article are publicly available. Summary statistics for the sleep traits are available here at: [http://sleepdisordergenetics.org/]. Summary statistics for the Lambert *et al*., 2013, AD GWAS are available at: [https://www.niagads.org/datasets/ng00036] and for the more recent Jansen *et al*., 2019, AD GWAS are available at: [https://ctg.cncr.nl/software/summary_statistics].

## Supplementary Data

Supplementary data are available at *IJE* online.

## Funding

This work was supported by a grant from the UK Economic and Social Research Council (ES/M010317/1) and a grant from the BRACE Alzheimer’s charity (BR16/028). Research reported in this publication was supported by the National Institute on Aging of the National Institutes of Health (under Award No. R01AG0488350. L.D.H. and E.L.A. are supported by fellowships from the UK Medical Research Council (xyaMR/M020894/1 and MR/P014437/1, respectively). R.C.R. is a Vice-Chancellors research fellow at the University of Bristol. S.E.J. is funded by the Medical Research Council (grant: MR/M005070/1). M.N.W. is supported by the Wellcome Trust Institutional Strategic Support Award (WT097835MF) and UK Medical Research Council (MR/M005070/1). A.R.W. and T.M.F. are supported by the European Research Council (grants: SZ-245 50371-GLUCOSEGENES-FP7-IDEAS-ERC and 323195). R.B. is funded by the Wellcome Trust and Royal Society (grant: 104150/Z/14/Z). G.H. is supported by the Wellcome Trust and the Royal Society (208806/Z/17/Z). E.L.A., R.C.R., K.H.W., G.H., L.D.H., D.A.L. and G.D.S. work in a unit that receives funding from the University of Bristol (MC_UU_00011/1 and MC_UU_00011/6). D.A.L. is an National Institute for Health Research Senior Investigator (NF-0616–10102). K.H.W. is funded by the Wellcome Trust Investigator Award (202802/Z/16/Z, Principal Investigator: Professor Nicholas J Timpson). The content of this paper is solely the responsibility of the authors and does not necessarily represent the official views of any of the funders.

## Supplementary Material

dyaa183_Supplementary_Data
