# Supplementary figures and images for: Is disrupted sleep a risk factor for Alzheimer’s disease? Evidence from a two-sample Mendelian randomization analysis
Source: Int J Epidemiol. 2020 May 11;50(3):817–28. doi: 10.1093/ije/dyaa183 (PMC8271193; doi:10.1093/ije/dyaa183)

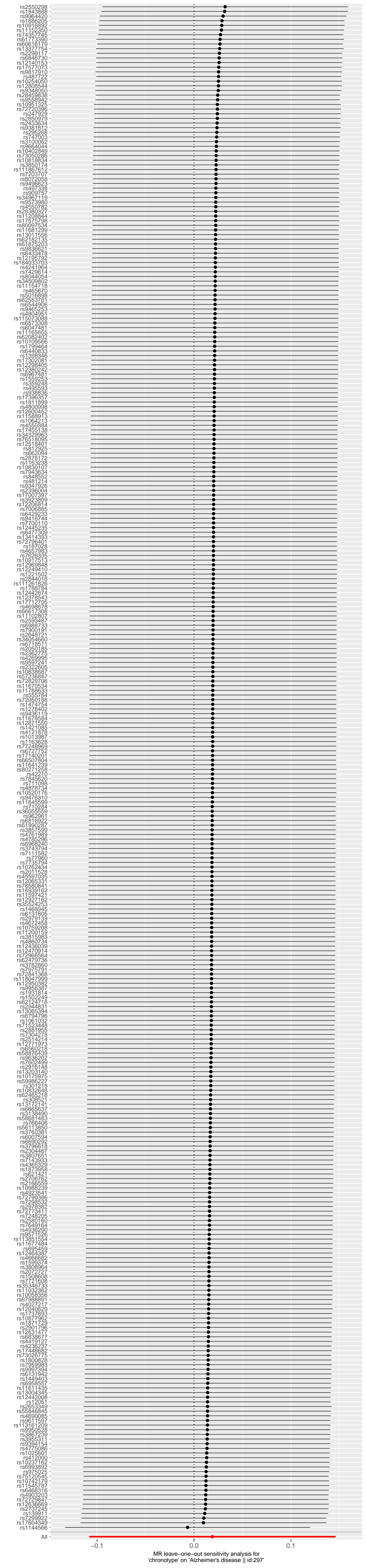

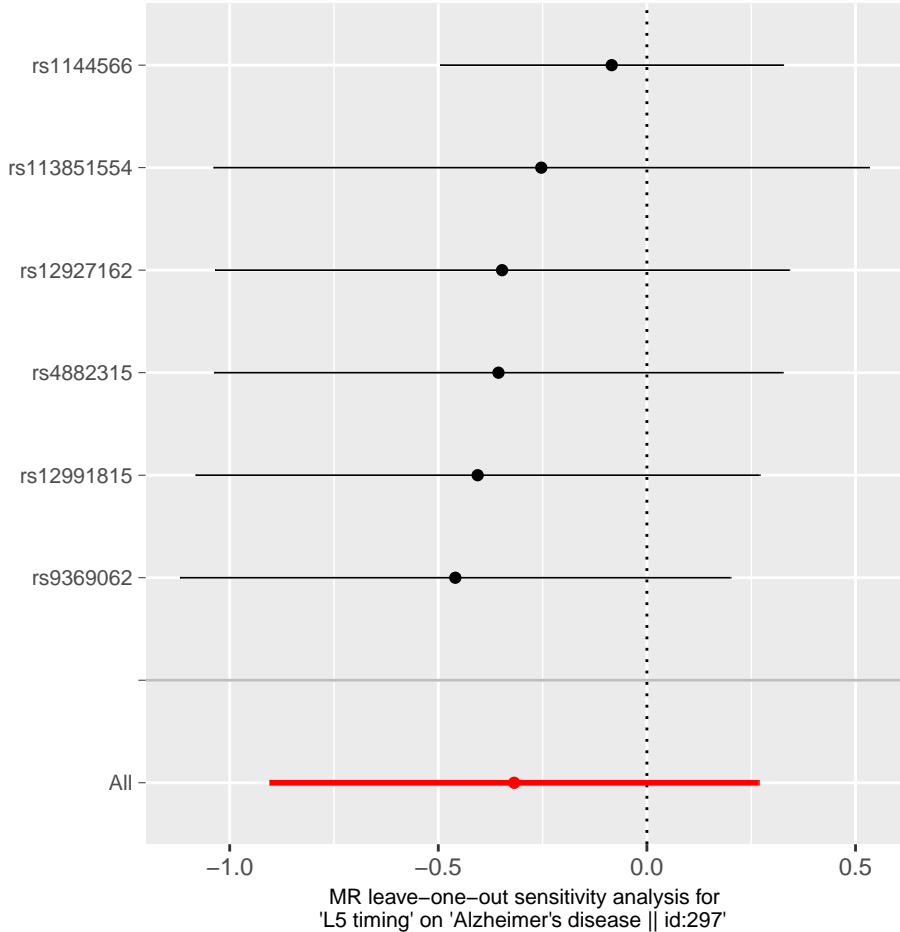

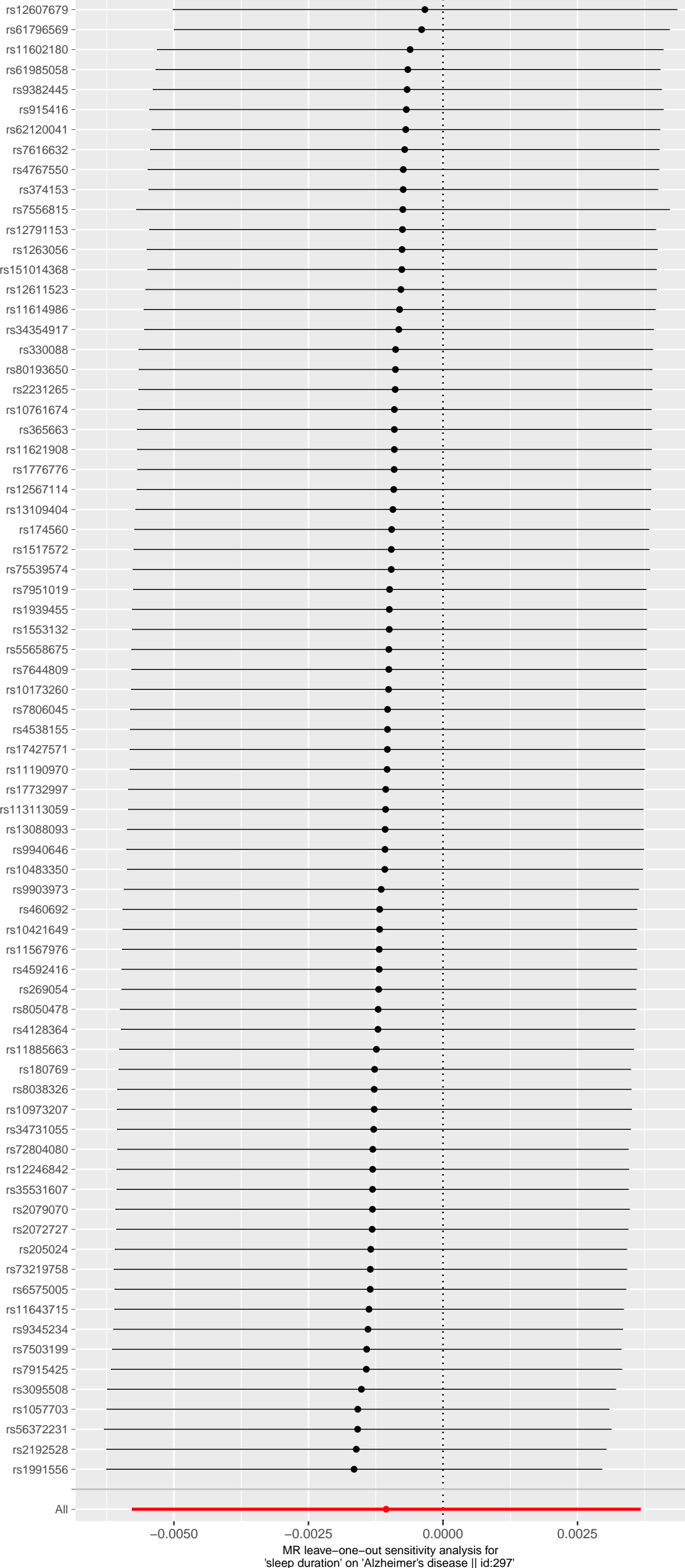

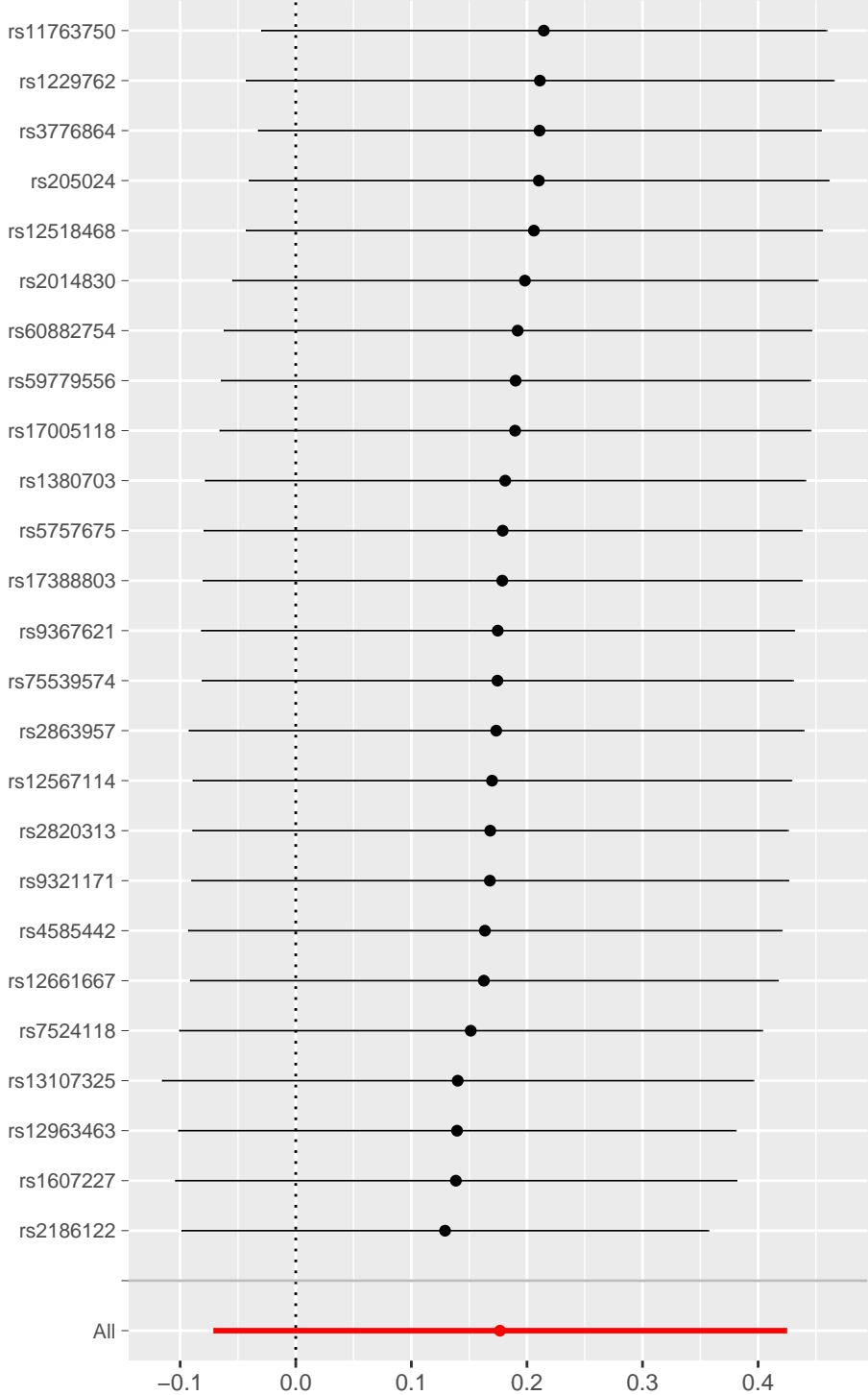

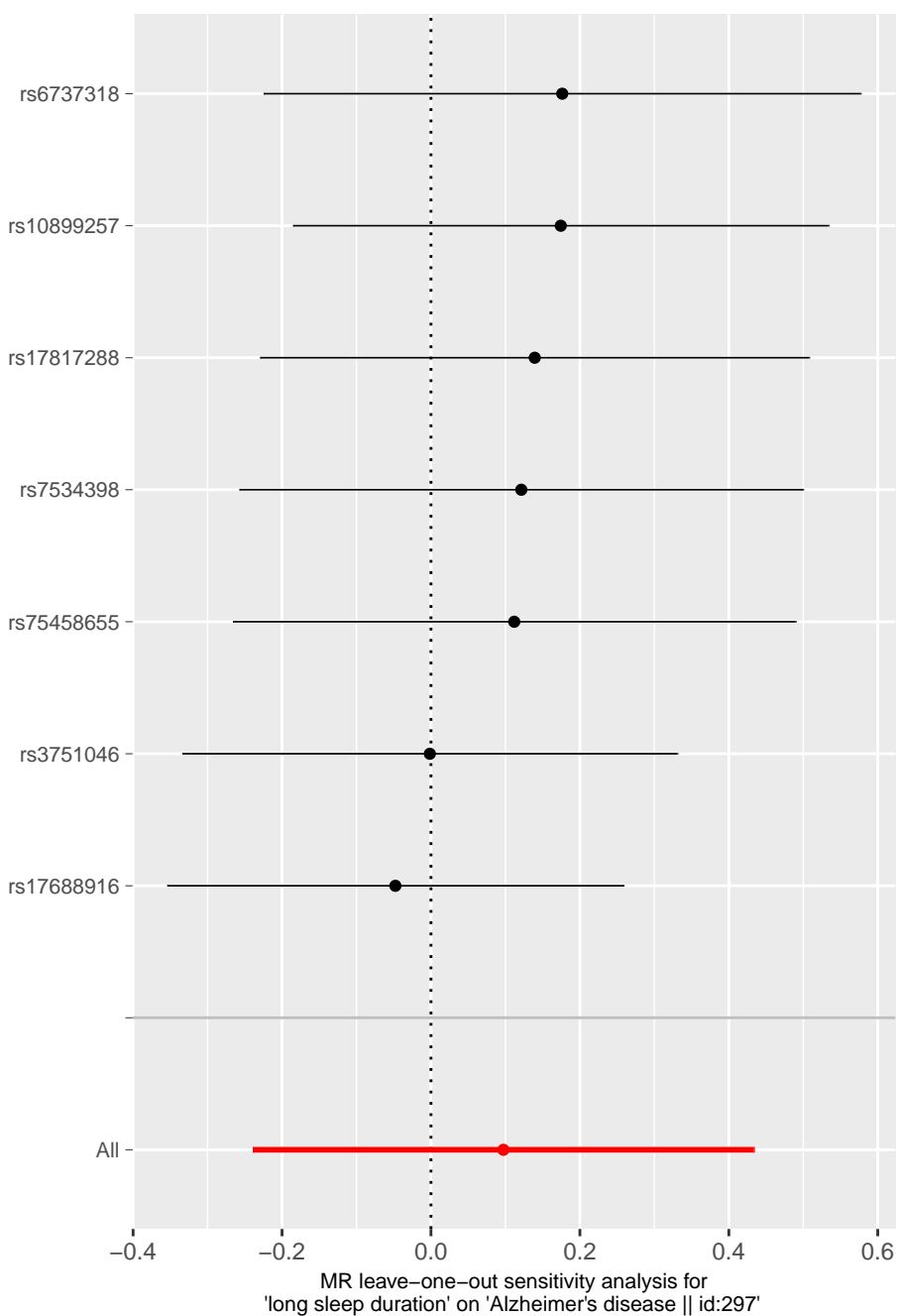

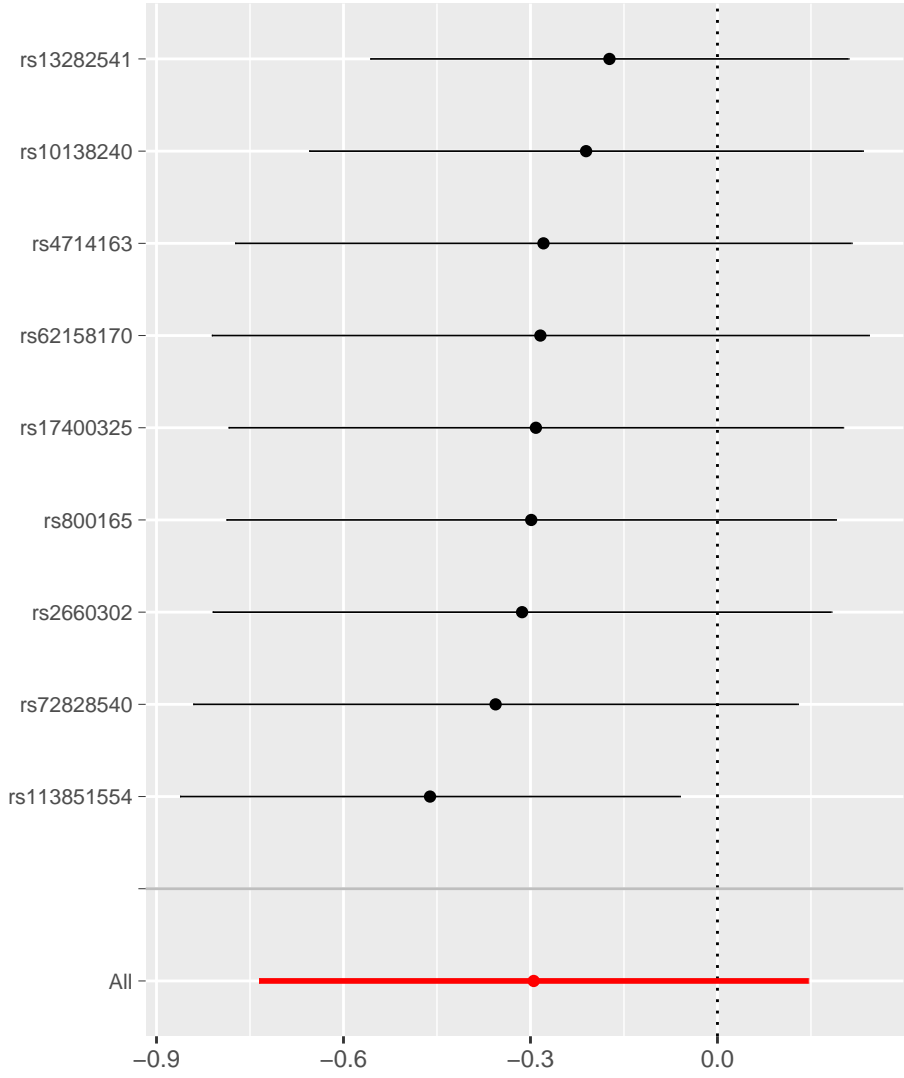

MR leave-one-out sensitivity analysis for 'sleep\_duration' on 'Alzheimer's disease || id:297'

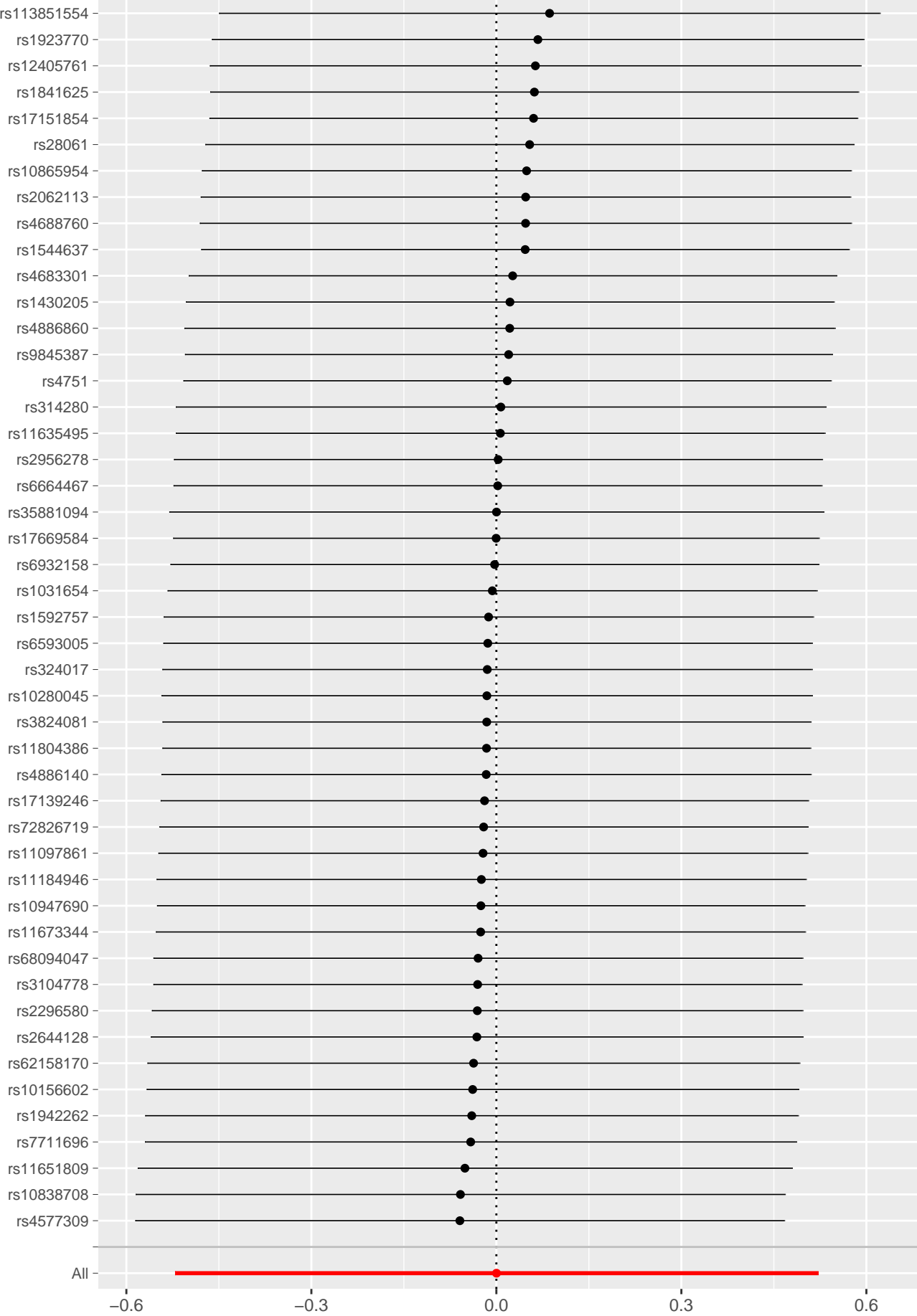

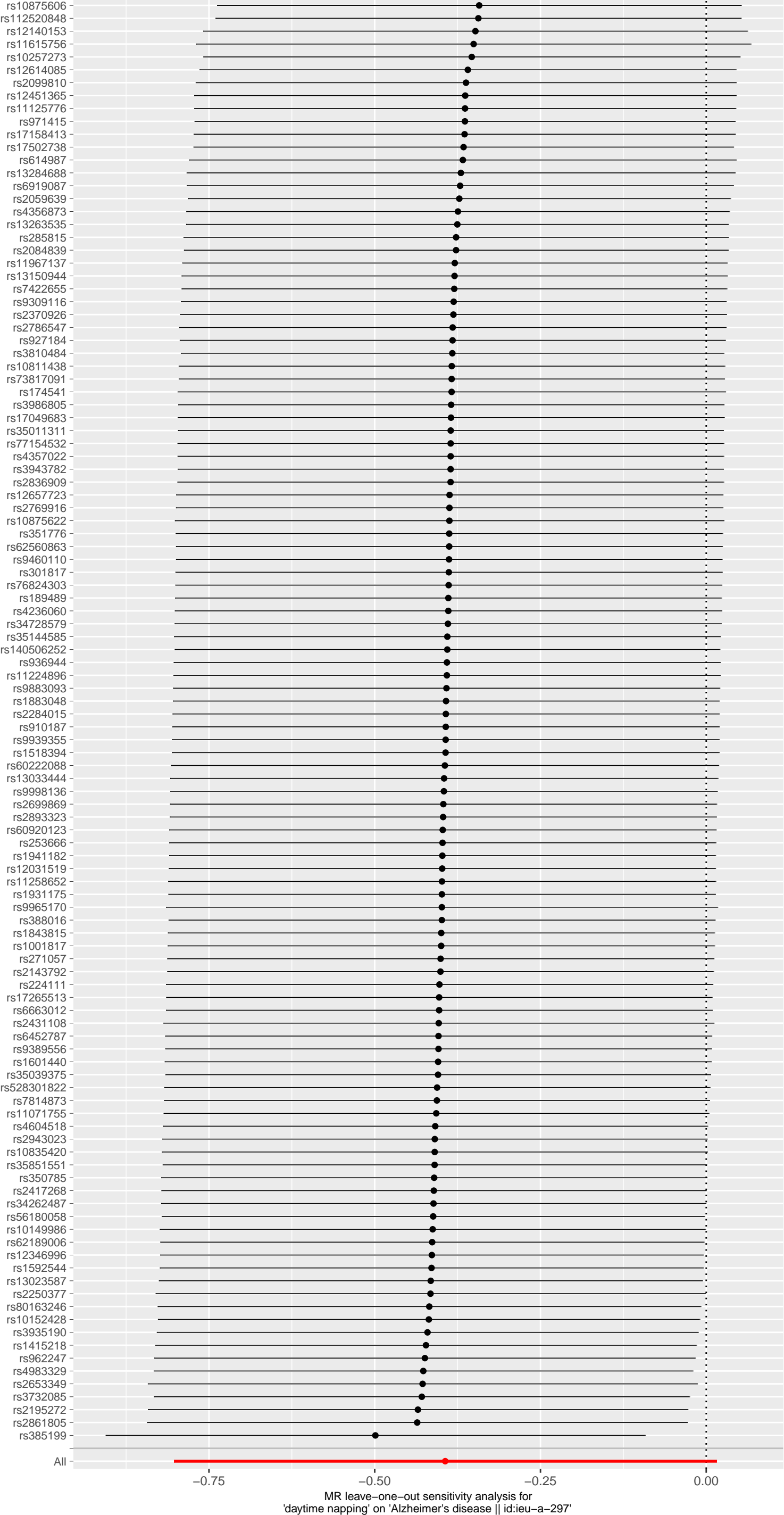

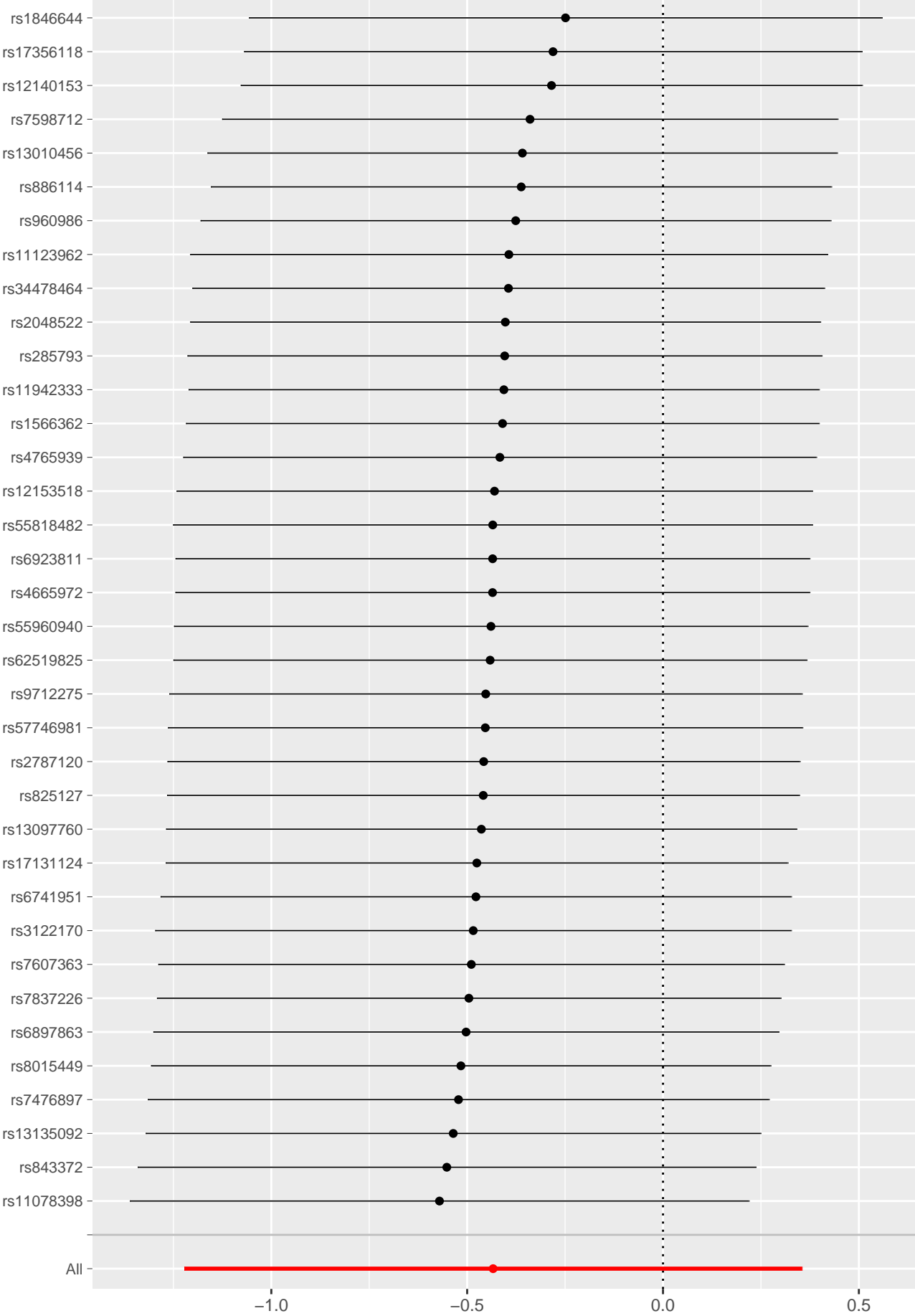

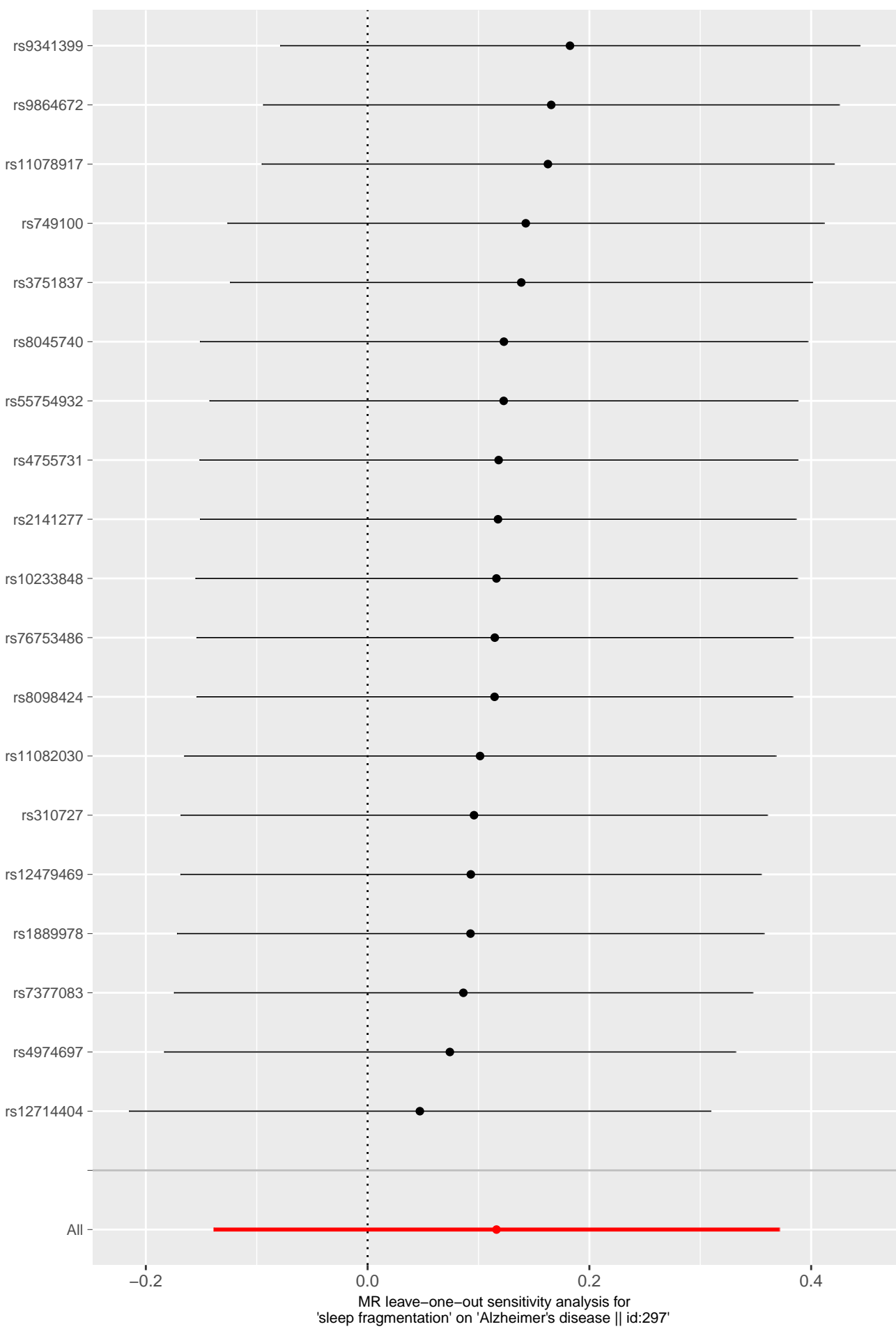

Supplement: dyaa183_Supplementary_Data [file dyaa183_supplementary_data.zip › ije-2019-11-1558-File008.pdf]
